# Neuronatin in a Subset of Glioblastoma Multiforme Tumor Progenitor Cells Is Associated with Increased Cell Proliferation and Shorter Patient Survival

**DOI:** 10.1371/journal.pone.0037811

**Published:** 2012-05-18

**Authors:** David S. Xu, Chunzhang Yang, Martin Proescholdt, Elisabeth Bründl, Alexander Brawanski, Xueping Fang, Cheng S. Lee, Robert J. Weil, Zhengping Zhuang, Russell R. Lonser

**Affiliations:** 1 Surgical Neurology Branch, National Institute of Neurological Disorders and Stroke, National Institutes of Health, Bethesda, Maryland, United States of America; 2 Brain Tumor and Neuro-Oncology Center, Cleveland Clinic Foundation, Cleveland, Ohio, United States of America; 3 Department of Neurosurgery, University Regensburg Medical Center, Regensburg, Germany; 4 Calibrant Biosystems, Gaithersberg, Maryland, United States of America; Wayne State University School of Medicine, United States of America

## Abstract

Glioblastoma multiforme is the most common and malignant primary brain tumor. Recent evidence indicates that a subset of glioblastoma tumor cells have a stem cell like phenotype that underlies chemotherapy resistance and tumor recurrence. We utilized a new “multidimensional” capillary isoelectric focusing nano-reversed-phase liquid chromatography platform with tandem mass spectrometry to compare the proteomes of isolated glioblastoma tumor stem cell and differentiated tumor cell populations. This proteomic analysis yielded new candidate proteins that were differentially expressed. Specifically, two isoforms of the membrane proteolipid neuronatin (NNAT) were expressed exclusively within the tumor stem cells. We surveyed the expression of NNAT across 10 WHO grade II and III gliomas and 23 glioblastoma (grade IV) human tumor samples and found NNAT was expressed in a subset of primary glioblastoma tumors. Through additional *in vitro* studies utilizing the U87 glioma cell line, we found that expression of NNAT is associated with significant increases in cellular proliferation. Paralleling the *in vitro* results, when NNAT levels were evaluated in tumor specimens from a consecutive cohort of 59 glioblastoma patients, the presence of increased levels of NNAT were found to be a an independent risk factor (P = 0.006) for decreased patient survival through Kaplan-Meier and multivariate analysis. These findings indicate that NNAT may have utility as a prognostic biomarker, as well as a cell-surface target for chemotherapeutic agents.

## Introduction

Glioblastoma Multiforme (GBM) is the most common and deadly (median survival of 1 year) adult central nervous system malignancy. Recent evidence demonstrates that a subset of GBM tumor cells harbor an undifferentiated stem cell-like phenotype and are refractory to conventional chemotherapeutic agents [1], [2]. To elucidate phenotypic markers of these tumor stem cells (TSCs), we previously utilized 2-dimensional polyacrylamide gel electrophoresis (2D-PAGE) for comparative proteomic analysis of GBM and normal brain tissue [3]. However, 2D-PAGE is significantly limited by its qualitative nature for detection of proteins in low cellular concentrations and the sample preparation requirements are incompatible with hydrophobic proteins including membrane bound proteins. These limitations preclude the identification of many candidate TSC markers, including membrane bound proteins that are potential chemotherapeutic targets.

To overcome the technical limitations of 2D-PAGE described above and to gain deeper insight into the potential TSC protein markers, we utilized a new method of “multidimensional” capillary isoelectric focusing nano-reversed-phase liquid chromatography with tandem mass spectroscopy (CIEF-nRPLC-MS) to compare the proteomes of primary TSC cultures before and after induction of differentiation with ciliary neurotrophic factor (CNTF) [4]. CIEF-nRPLC-MS permits a 15-fold increase in protein identification compared to 2D-PAGE and is fully compatible with preparations isolating hydrophobic proteins. Subsequently, application of CIEF-nRPLC-MS permits the highly sensitive and reliable identification and quantitative comparison of proteins between different isolated cell populations.

Using CIEF-nRPLC-MS, we isolated a number of TSC membrane proteins that showed at least a 3 log-fold difference in expression after differentiation. Among these, both α and β isoforms of the proteolipid, neuronatin (NNAT), were found to be consistently expressed in TSCs but non-existent within their differentiated counterparts. Given the putative involvement of NNAT with the embryological development of the CNS, as well as reports of its association with more aggressive phenotypes in medulloblastoma [5]–[7], we sought to confirm the proteomic finding of upregulated NNAT in GBM TSCs and to establish its utility as a prognostic biomarker in GBM patients. We then addressed its putative biological role and potential signaling mechanisms through in vitro transfection of the human glioma cell line U87 with fluorescent protein tagged constructs.

Our findings indicate that NNAT is endogenously expressed at low levels in normal human brain tissue, but becomes over expressed in a subset of primary GBM tumors. When transfected into U87 glioma cells, both isoforms of NNAT were associated with significant increases in cellular proliferation. Furthermore, high levels of NNAT expression were correlated to significantly shorter overall survival in a separate cohort of GBM patients. The molecular mechanisms underlying this effect have yet to be elucidated, but given NNAT's expression pattern, it may play a specific role in the pathogenesis or maintenance of primary GBM TSCs.

## Results

### CIEF-nRPLC-MS identifies differential NNATα and NNATβ expression in undifferentiated and differentiated TSCs

TSCs were established from human GBM tissue. Cultured cells were maintained in an undifferentiated state, confirmed by positive immunocytochemical staining for the neural stem cell markers Nestin and Sox2. Differentiation of TSCs was induced by treatment with CNTF and demonstrated by loss of Nestin and Sox2 expression, as well as concomitant gain of GFAP expression as previously described [3].

Both undifferentiated and CNTF-treated differentiated glioma TSCs were analyzed using CIEF-nRPLC-MS/MS. Peptide identification was based on 3 runs of a single tissue sample and was limited by high-mass-accuracy (60 ppm) and high-confidence (5% false-positive) hits to fully tryptic proteins. Expression differences between the undifferentiated and the differentiated TSC proteomes yielded 174 membrane proteins. Among those proteins, both NNATα and NNATβ were identified to have greater than 3-log fold levels in TSCs prior to differentiation with near undetectable levels found in the differentiated cells.

### NNAT is expressed in a subset of normal glial cells

**Figure 1:**
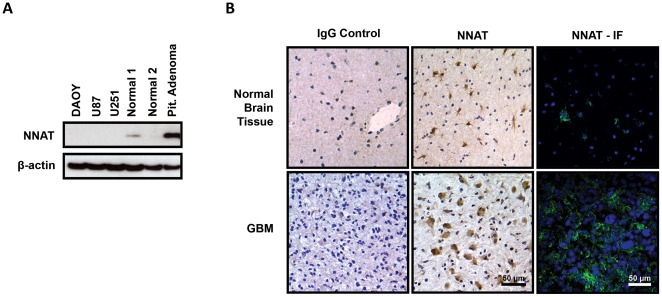
Validation of NNAT Antibody. We selected a commercial polyclonal antibody (abcam, ab30332) targeting the C-terminus of both NNAT isoforms for use in our study. To verify its specificity, we probed several tumor cell lines, normal brain, and pituitary adenoma samples as a positive control through western blot (A) and combined immunohistochemistry and immunofluoresence (B). The antibody was specific in identifying a band corresponding to the molecular weight of NNAT within positive control samples. Normal brain tissue exhibited endogenous NNAT expression in a subset of astrocytes. A small proportion of GBMs strongly stained for NNAT with cytoplasmic and membrane localization.

To assess the efficiency and specificity of a commercial antibody derived against NNAT, we used western blotting to probe for NNAT in brain tumor derived cell lines (U87 and U251), normal brain tissue and tumor resection specimens (Figure 1A). NNAT expression was found to be absent in the medulloblastoma cell line DAOY, as well as the two glioma derived cell lines. NNAT was found to have low expression in normal brain, but was abundant within pituitary adenoma, a known positive control [8]. We further confirmed our western blot findings with immunohistochemistry and immunofluorescent staining (Figure 1B). Consistent with western blot data, normal brain tissue exhibited a small population of cells that express NNAT. These cells commonly exhibited glial morphology. Contrarily, NNAT expression in GBM samples yielded populations of cells that stained richly for NNAT.

### NNAT is overexpressed without mutation in a subset of primary GBMs with EGFR expansion

**Figure 2:**
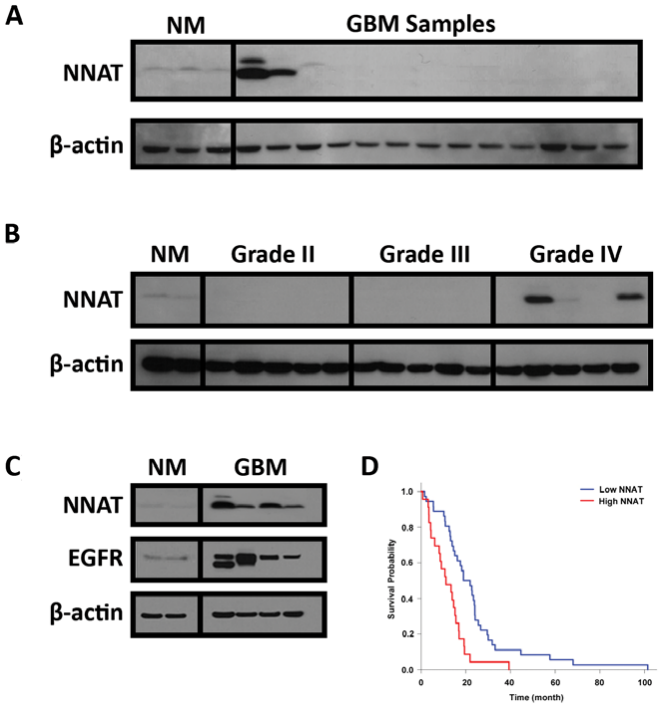
NNAT is Expressed in a Subset of Primary GBMs. (A) We initially surveyed 13 GBM samples for NNAT expression and found higher than baseline levels in 2 of the samples. (B) NNAT expression was not found in 10 WHO Grade II and III tumors, but was positive in an additional 5 GBM samples. A total of 23 GBM samples were assayed that overexpression of NNAT was found in 4 (17%) of the samples. (C) All tumors that expressed NNAT also had concurrent upregulation of the EGFR receptor, a feature that is associated with primary GBMs that arise de novo.

After validating the NNAT antibody, we sought to assess the prevalence of NNAT expression within resected GBM samples. We initially evaluated 13 GBMs and identified NNAT expression through western blot analysis (Figure 2A). We found 2 out of 13 GBM samples showed NNAT expression above the levels within normal tissue. We next performed western blot analysis on a series of gliomas with different WHO pathologic grades (Figure 2B). NNAT was found to be absent in WHO Grade II (5 out of 5) and Grade III (5 out of 5) gliomas, but was expressed higher than normal baseline levels in 2 out of 5 cases of WHO Grade IV gliomas (GBM). A total of 23 GBMs were assayed for the presence of NNAT, and 4 of them were found to have increased NNAT expression compared to normal brain tissue. Clinical patient data verified that these tumors had presented as de novo lesions with a pathologic diagnosis of WHO Grade IV at their onset.

The absence of NNAT expression within lower grade astrocytomas (Grade II and III) prompted us to question whether its expression is limited to primary de novo GBMs. Subsequently, we assessed for over expression of EGFR, a finding strongly associated with primary GBMS, among NNAT positive tumors and found all of them to be strongly positive for EGFR over expression (Figure 2C). We then questioned whether the aberrant expression of NNAT might be due to a genetic mutation within the NNAT genome and sequenced the coding regions of the NNAT gene in all 23 GBM samples. None of the tumors were found to harbor any exonal mutations.

### High NNAT expression is associated with poor survival in GBM patients

A summary of patient characteristics in the survival analysis is detailed in Table 1. Among the 59 patients studied, 23 harbored a tumor with high NNAT expression. Median survival time for patients with high NNAT expression was 11.1 months, in contrast to 18.9 months in patients with low NNAT expression. This difference is statistically significant in univariate analysis (P<0.001, Figure 2D). Results of the multivariate analysis using the Cox proportional hazards model is detailed in Table 2, and revealed that high NNAT is an independent prognostic factor indicating shorter overall survival (P = 0.006). In addition to NNAT expression, older age and low Karnofsky score were additional factors associated with poor prognosis, whereas the extent of resection was not found to be a significant prognostic factor in this patient population.

**Table 1:** Patient demographics.

	NNAT high	NNAT low	ρ
Total Patients	23	36	
Male/Female	16/7	12/13	0.22
Median Age	63.4	56.1	0.06
Median KPS	90	90	0.66
GTR (%)	31.0	34.8	0.96

**Table 2:** Cox Proportional Hazards Data.

	Hazard Ratio	95% CI		ρ	Standard Error
		Low	High		
NNAT (+)	7.44	1.17	2.64	0.006	0.21
Age	6.32	1.00	1.04	0.012	0.11
KPS	3.87	6.5	10.0	0.049	1.58
Extent of resection	0.07	1.00	2.00	0.798	0.06

### NNAT expression drives cellular proliferation

**Figure 3:**
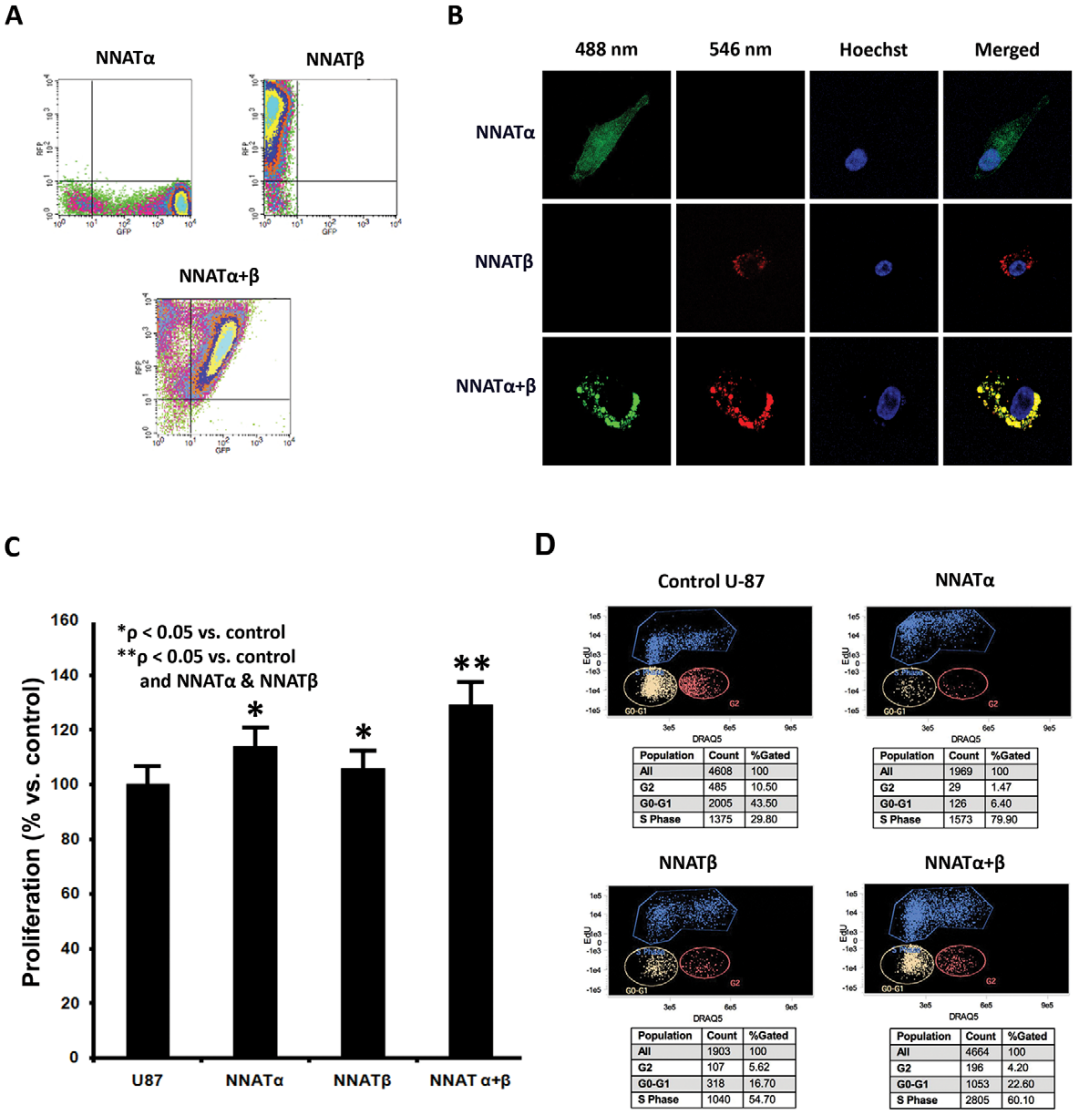
Expression of NNAT is Associated with Increased Cellular Proliferation. GFP-tagged NNATα and RFP-tagged NNATβ were transfected alone or in conjunction into U87 glioma cells. The cells were grown in antibiotic selection media utilizing G418 and underwent FACS (A) repeatedly over the course of 5 weeks to select a stably transfected population with at least 2 log-fold increase in fluorescent protein expression compared to controll. (B) The cellular distribution of NNATα was predominantly membrane bound, with some cytoplasmic localization as well. NNATβ was more strongly localized in the cytoplasm, and when coexpressed, the two isoforms were found to strongly co-localize with NNATα expression moving more into the cytoplasm. (C) Utilizing a colorimetric MTT proliferation assay, expression of either form of NNAT dramatically increased the proliferation profile of the U87 cells versus control. Coexpression of both isoforms caused an increase in proliferation that was greater than expression of either isoform alone. (D) Using a fluorescent activated cell sorting based proliferation demonstrated high percentage of S-phase cells in the NNATα (79.9%) and NNATβ (54.7%) cell lines versus control (29.8%). Coexpression of both NNAT isoforms also yielded a higher population of S-phase cells (60.1%).

The high expression of NNAT limited to a subset of primary GBMs indicates its potential functional impact in tumor growth and cell proliferation. Prior studies have found NNAT to be a potent stimulator of cellular proliferation in medulloblastoma cells [5]. To determine if NNAT plays an analogous role within gliomas, we established U87 malignant glioma cell lines with stable NNAT expression. The constructs of the two spliced forms of NNAT, NNATα-EGFP and/or NNATβ-DsRed were delivered into U87 malignant glioma cells and selected through two rounds of fluorescent assisted cell sorting (Figure 3A). Confocal microscopy revealed that NNATα (green) was expressed homogeneously in the cytoplasm of U87 cells, whereas NNATβ (red) located as punctuated aggregation around the nucleus (Figure 3B). When co-expressed in the same cell, NNATα and NNATβ were found to be co-localized as punctuated structures in the cytoplasm, indicating potential heterodimer interactions.

The impact of NNAT on tumor cell growth was first assessed through the MTT proliferation assay (Figure 3C), in which NNATα and NNATβ increased cell proliferation by 13.8% and 5.8%, respectively (P<0.05 vs. control), with coexpression of both isoforms enhancing proliferation by 29.2% (P<0.01 vs. control and single isoforms). Further analysis through EdU-derived high content cell cycle screening revealed dramatic changes in the population of S-phase cells across the cells lines (Figure 3D). The percentage of S-phase cells in the control U87 MG line was 29.8%, while in the NNATα and NNATβ transfected lines, the percentage rose to 79.9% and 54.7%, and in the co-expression line it increased to 60.1%.

## Discussion

Using our high throughput proteomic technique, CIEF-nRPLC-MS, we identified a number of novel differentially expressed membrane proteins in GBM TSCs versus their differentiated counterparts. NNAT was identified and selected for further investigation due to its previously described role in embryologic CNS development as well as its association with more aggressive phenotypes of medulloblastoma [5], [9]. When surveyed across 23 different GBM tumors, NNAT showed elevated baseline expression in 4 of them (17%). More interestingly, NNAT expression was not found in any of the lower WHO grade II and III astrocytomas (Fig. 2). In a larger cohort, we found that high NNAT expression is associated with poor survival in GBM patients. This might be due to the biological function of NNAT itself, promoting cell proliferation and tumor growth, or simply reflecting a higher abundance of NNAT expressing GBM TSCs in these more aggressive tumors. This latter finding may correlate with a recent distinction made between primary and secondary GBMs based on the sequential genetic aberrations that occur in tumors presenting in two separate patient populations [10], [11]. Primary GBMs present in older patients with a mean age greater than 50 years old and are characterized by overexpression of a mutated form of epidermal growth factor receptor (EGFR), deletions in the phosphatase and tensin homolog (PTEN) gene in chromosome 10, and p16 deletion [12]–[14]. Secondary GBMs occur in younger patients and present as a recurrence of an earlier lower WHO grade tumor that has undergone malignant progression. These tumors typically show overexpression of the platelet derived growth factor receptor (PDGFR), mutations within p16, and aberrations within the p53 gene [12], [15]. Within secondary GBMs, the accumulation of these mutations is seen early in the malignant progression of the tumor, with p53 mutations as well as PDGFR over-expression seen in >60% of WHO grade II and III astrocytomas [11]. The presence of NNAT expression in none of the lower grade tumors, suggests that it may play a distinct role in the pathobiology of primary GBMs, specifically within the recently characterized EGFR genetic subtype, and excluded from the NF1 and PGFRA/IDH1 subtypes [16].

Current knowledge regarding the functional role of NNAT is still limited. Structurally, NNAT belongs to the proteolipid family of proteins and is thought to be membrane associated due to having an N-terminus that is heavily enriched with hydrophobic amino acids. Multiple studies examining other cell types have found NNAT localization to be robust not only on the cell membrane, but also in the cytoplasm as well [17]–[19]. The significance of this compartmentalization is unknown, but such a distribution may reflect vesicular trafficking of NNAT or its active interplay with other signal transduction intermediaries. In humans, NNAT is alternatively spliced with an α and β isoform that differ by a 81-bp transmembrane sequence in the middle of the coding region present within the α isoform. Human NNAT mRNA is most abundantly expressed in the human fetal brain at 18–24 weeks of development, and undergoes down-regulation during adulthood except in the anterior pituitary gland [8]. In mice, NNAT expression also occurs early in fetal development, with NNATα expression preceding and ending sooner than the appearance of NNATβ [9]. Spatially within the fetal mouse brain, NNAT expression is first seen specifically within rhombomeres 3 and 5, the origins of the hindbrain, prior to becoming generalized throughout the CNS [6]. The spatial and temporal pattern of NNAT expression suggests that it participates in segment identity of the developing mammalian brain, as well as maintenance of developing as well as post-mitotic neuroepithelial cells.

Previous studies in medulloblastoma show that expression of both NNAT isoforms is associated with increased proliferation and anchorage independent growth, but that the presence of NNATα is the primary determinate of cytologic response [5], [20]. Our results parallel this finding within U87 glioma cells, showing that expression of both NNATα and NNATβ is associated with increased U87 proliferation by MTT assays as well as an increased proportion of cells active in S-phase (Fig. 3 C, D). We also found the proliferative effects of NNATα to be greater than NNATβ, but co-expression of both isoforms produced a greater than additive effect seen in the MTT proliferation assay that could not be explained by cell-cycle dependence (Fig. 3C). One possible explanation is that the expression of both isoforms not only increases cellular proliferation, but also enhances cellular viability in regards to anchorage-dependent growth and other culture stresses. Furthermore, this additive effect suggests that the two NNAT isoforms may interact, which is further supported by our finding that the cellular distribution of NNATα becomes more cytoplasmic when coexpressed with NNATβ and that the two strongly colocalize (Fig. 3B). The signal transduction pathway that mediates these proliferative responses is currently unknown. However, other members of the proteolipid family of proteins function as modulatory subunits of ion channels with C-termini that can serve as adaptor sites for other regulatory and signaling proteins [7], [21].

The evidence we have accumulated suggests that NNAT is present within normal human brain tissue limited to a specific population of astrocytes (Fig. 1), is overexpressed in a subset of primary GBM (Fig. 2), and strongly corresponds with elevated *in vitro* proliferation (Fig. 3 C, D). It is tempting to hypothesize that those astrocytes may be the very antecedent cells that undergo malignant transformation and or dedifferentiation to form GBM TSCs. However, we were not able to identify any mutations in the NNAT coding region from our 23 GBM tumor samples that could act as a trigger for this aberrant expression. Other possibilities that cannot be excluded at this time are intronic regulatory unit mutations, increased upstream signaling, or loss of epigenetic regulation. Given the limited subset of GBMs that NNAT is expressed within, it is unlikely to be a primary driver of GBM pathogenesis, but rather may be secondarily induced through a bystander effect due to derailment of other molecular signaling pathways. However, given what little is known about NNAT, especially with regard to primary CNS malignancies, it is a promising target for further study that is likely to shed light on novel signaling pathways involved in GBM pathobiology as well as potential therapeutic targets for intervention.

## Materials and Methods

### Clinical materials

All tissue samples and clinical information were obtained as part of an institutional review board-approved repository program at the Surgical Neurology Branch at the National Institute of Neurological Disorders and Stroke at the National Institutes of Health, the Brain Tumor Institute at the Cleveland Clinic Foundation and the Department of Neurosurgery at the University of Regensburg Medical Center. For this study, the authors obtained IRB approval at their respective institutions to use both fresh frozen glioma tissue specimens as well as normal human brain tissue. Tumor samples were evaluated by the respective pathology department of their originating institution to ensure proper WHO tumor diagnosis, grading, as well as exclusion of samples taken from tumor margins with large quantities of normal brain tissue. Informed consent for usage of these samples was waived by each respective IRB on the basis of adherence to the ‘Privacy Rule’, where all work utilized tissues from an existing repository and that usage of research specimens posed no more than a minimal risk to the privacy of the sample donors.

### Patient population

For the survival studies, clinical data and tumor specimens from a cohort of 59 consecutive patients (20 female, 39 male) treated at the University of Regensburg Medical Center were investigated. Mean age of the patients was 59.5 years (range, 33.6 to 82.5 years), the median Karnofsy Performance Score was 90, (range, 50 to 100). All patients underwent initial craniotomy for tumor resection. After resection, patients received combined adjuvant treatment consisting of external beam radiation and chemotherapy with temozolomide according to the Stupp regimen [22].

### GBM TSC line and cell lines

Primary TSC cultures from freshly procured human GBM tumors were obtained and expanded as previously described [3]. TSCs were grown in poly-d-lysin/laminin coated dishes with DMEM/F12 medium (Invitrogen; Carlsbad, CA) supplemented with N-2 (0.5×; Invitrogen), B-27 without vitamin A (0.5×; Invitrogen), fibroblast growth factor (20 ng/ml; Invitrogen), and epidermal growth factor (20 ng/ml; R&D Systems) that was changed daily. Induction of TSC differentiation was carried out by addition of ciliary neurotrophic factor (20 ng/ml; R&D Systems). Human medulloblastoma cell line DAOY, glioma cell lines U87 MG and U251 were maintained in Dulbecco's Modified Eagle Media (DMEM, Invitrogen) containing 10% FBS, 100 U/mL penicillin and 100 µg/mL streptomycin.

### CIEF-nRPLC-MS proteomic profiling

Samples of TSCs prior to and after treatment with CNTF were harvested for proteomic profiling by Calibrant Biosystems (Gaithersburg, MD), as previously described [23]. Briefly, collected cells were digested by trypsin, and fractionated into 12 fractions by CIEF. The fractions were then sequentially analyzed through nRPLC and fed into a quadrupole time-of-flight micro mass spectrometer (Waters, Milford, MA). Peptide and protein identifications were then made using MASCOT 2.0 (Matrix Science, London, UK) utilizing a reversed proteome database search approach. The incidence of unique tryptic peptides were then used to assess relative quantities of their corresponding protein and a comparison of proteomic profiles between native and differentiated TSCs was made.

### Western blot and antibodies

Microdissected tissue and cell pellets were collected and lysed in T-PER lysis buffer (Thermo), sonicated, and centrifuged. The quantity of protein was determined using a colorimetric Bio-Rad Protein Assay Kit (Bio-Rad). Proteins were separated on NuPAGE 4 to 12% Gradient Bis-Tris gels (Invitrogen) and transferred to PVDF membranes. Membranes were blocked in 5% skim milk in TBS with 0.05% Tween-20 and probed with primary antibody. Visualization was carried out through a species specific horseradish peroxidase conjugated secondary antibody system paired with the Pierce SuperSignal West Pico enhanced chemilumescent substrate (Thermo; Rockford, IL).

Antibodies used in this study: EGFR (4267S, Cell Signaling Technology), GFAP (3670S, Cell Signaling Technology), Nestin (ab22035, Abcam), NNAT (ab27266, Abcam), β-Actin (sc-47778, Santa Cruz), Sox2 (3579S, Cell Signaling Technologies).

### Immunostaining

Cells or tissue slices were fixed in Histochoice H108 (Mandel; Guelph, Ontario, Canada) and incubated in 0.3% Triton X-100/TBS before being blocked with 5% BSA/TBS and subsequently labeled with anti-NNAT (1∶100, Abcam) antibodies overnight. For immunofluorescence studies, visualization utilized a species specific Alexa Fluor conjugated secondary antibody (Sigma) followed by nuclear counterstaining with Hoechst 33342 (Invitrogen) before examination using a Zeiss LSM510 confocal microscope. Immunohistochemistry studies utilized a species specific horseradish peroxidase conjugated secondary antibody paired with the VECTASTAIN ABC System (Vector Laboratories, Burlingame, CA) for colorimetric detection. The specimens were then examined using a Leica DM LB light microscope. Grading of immunohistochemical staining was performed by three observers who were blinded to the clinical outcome of the patients. The rating of the immunohistochemical staining was performed by using a four-grade, semiquantitative scale (1, no cells positive; 2, 1 to 10% positive; 3, 10 to 30% positive; 4, more than 30% positive). High NNAT expression was defined by an average rating higher than grade 3 for each patient.

### NNAT Sequencing

DNA extraction from human GBM tumors was performed using the Qiagen DNeasy Blood and Tissue Kit (Qiagen, Valencia CA). Genomic DNA was utilized as a template for polymerase chain reaction targeting the 3 coding regions of NNAT. The primers are as follow: Exon 1 ( Forward – 5′TTTCTCGACCACCCACCTAC; Reverse – 5′CGGCAATCGGAATAGCAC), Exon 2( Forward – 5′TGCCAAAGGAATCGCATATT; Reverse – 5′GCTGATTGGACCCACAACTT), Exon 3( Forward – 5′GTGGGTGCTCTCCACTAAGG, Reverse – 5′AGGAGCACCTGATGATACGG). Sequencing was subsequently performed at the NINDS DNA Sequencing Facility and results were aligned and analyzed using the ClustalW2 program available online.

### Stable Transfection of U87 MG Cells

The full-length human cDNA of the NNATα and NNATβ isoforms were inserted into pCMV6 mammalian expression vectors. To distinguish the two transcriptional variants, NNATα was fused with an EGFP tag, whereas NNATβ was fused with a DsRed tag. Twenty-four µg of NNATα-EGFP or NNATβ-DsRed vector was delivered into 1.0×10^7^ U87 MG cells with lipofectamine 2000 reagent (Invitrogen). Media was replaced on the fifth day with selection media containing 400 µg/mL G418 and the lines were maintained in selection media for 2 weeks. Stable clones were isolated through three rounds of fluorescence-activated cell sorting (FACS) as previously described [24]. Cells with >95% purity were maintained in DMEM containing 10% FBS, 100 U/mL penicillin and 100 µg/mL streptomycin for subsequent analysis.

### Proliferation and Cell Cycle analysis

To visualize cell cycle changes, stable transfected U87 MG cells were labeled with 20 µM EdU (Invitrogen) for 4 hours. Cells were then labeled using Click-iT EdU HCS assay kit with either Alexa Fluor 488 or pacific blue labeling. DNA was counterstained with DRAQ5 (5 µM, Cell Signaling Technology). Cells were analyzed using an Image Stream X flow cytometry system (Amnis). IDEAS software was used to acquire and quantify fluorescent signal intensities and to graph the data as bivariate dot density plots for cell cycle determination.

### Statistics

Comparative significance testing of continuous variables was performed using the students t-test with a significance threshold defined at P<0.05. For the clinical analysis, survival time was measured from the date of resection until death of the patients. Survival rate was calculated by the Kaplan–Meier method and comparisons of survival between the different groups were estimated by generalized Wilcoxon test for univariate analysis. For multivariate analysis, Cox's proportional hazards model (forward stepwise procedure) was used. In addition to NNAT expression, age, Karnofsky score, and the extent of resection were analyzed as potential prognostic factors(SPSS Version 17.0, SPSS Inc., Chicago IL).

## References

[1] Singh SK, Hawkins C, Clarke ID, Squire JA, Bayani J (2004). Identification of human brain tumour initiating cells.. Nature.

[2] Bao S, Wu Q, McLendon RE, Hao Y, Shi Q (2006). Glioma stem cells promote radioresistance by preferential activation of the DNA damage response.. Nature.

[3] Park DM, Li J, Okamoto H, Akeju O, Kim SH (2007). N-CoR pathway targeting induces glioblastoma derived cancer stem cell differentiation.. Cell Cycle.

[4] Wang Y, Balgley BM, Rudnick PA, Evans EL, DeVoe DL (2005). Integrated Capillary Isoelectric Focusing/Nano-reversed Phase Liquid Chromatography Coupled with ESI–MS for Characterization of Intact Yeast Proteins.. Journal of Proteome Research.

[5] Siu IM, Bai R, Gallia GL, Edwards JB, Tyler BM (2008). Coexpression of neuronatin splice forms promotes medulloblastoma growth.. Neuro Oncol.

[6] Wijnholds J, Chowdhury K, Wehr R, Gruss P (1995). Segment-specific expression of the neuronatin gene during early hindbrain development.. Dev Biol.

[7] Dou D, Joseph R (1996). Cloning of human neuronatin gene and its localization to chromosome-20q 11.2-12: the deduced protein is a novel “proteolipid’.. Brain Res.

[8] Usui H, Morii K, Tanaka R, Tamura T, Washiyama K (1997). cDNA cloning and mRNA expression analysis of the human neuronatin. High level expression in human pituitary gland and pituitary adenomas.. J Mol Neurosci.

[9] Joseph R, Dou DX, Tsang W (1994). Molecular Cloning of a Novel mRNA (Neuronatin) That Is Highly Expressed in Neonatal Mammalian Brain.. Biochemical and Biophysical Research Communications.

[10] Ohgaki H, Kleihues P (2007). Genetic pathways to primary and secondary glioblastoma.. Am J Pathol.

[11] Wen PY, Kesari S (2008). Malignant Gliomas in Adults.. New England Journal of Medicine.

[12] Watanabe K, Tachibana O, Sata K, Yonekawa Y, Kleihues P (1996). Overexpression of the EGF receptor and p53 mutations are mutually exclusive in the evolution of primary and secondary glioblastomas.. Brain Pathol.

[13] Tohma Y, Gratas C, Biernat W, Peraud A, Fukuda M (1998). PTEN (MMAC1) Mutations Are Frequent in Primary Glioblastomas (de novo) but not in Secondary Glioblastomas.. Journal of Neuropathology & Experimental Neurology.

[14] Ueki K, Ono Y, Henson JW, Efird JT, von Deimling A (1996). CDKN2/p16 or RB Alterations Occur in the Majority of Glioblastomas and Are Inversely Correlated.. Cancer Research.

[15] Kleihues P, Ohgaki H (1999). Primary and secondary glioblastomas: From concept to clinical diagnosis.. Neuro-Oncology.

[16] Verhaak RGW, Hoadley KA, Purdom E, Wang V, Qi Y (2010). Integrated Genomic Analysis Identifies Clinically Relevant Subtypes of Glioblastoma Characterized by Abnormalities in PDGFRA, IDH1, EGFR, and NF1.. Cancer Cell.

[17] Revill K, Dudley KJ, Clayton RN, McNicol AM, Farrell WE (2009). Loss of neuronatin expression is associated with promoter hypermethylation in pituitary adenoma.. Endocrine-Related Cancer.

[18] Vrang N, Meyre D, Froguel P, Jelsing J, Tang-Christensen M (2010). The Imprinted Gene Neuronatin Is Regulated by Metabolic Status and Associated With Obesity.. Obesity.

[19] Chu K, Tsai M-J (2005). Neuronatin, a Downstream Target of BETA2/NeuroD1 in the Pancreas, Is Involved in Glucose-Mediated Insulin Secretion.. Diabetes.

[20] Yokota N, Mainprize TG, Taylor MD, Kohata T, Loreto M (2004). Identification of differentially expressed and developmentally regulated genes in medulloblastoma using suppression subtraction hybridization.. Oncogene.

[21] Kagitani F, Kuroiwa Y, Wakana S, Shiroishi T, Miyoshi N (1997). Peg5/Neuronatin is an imprinted gene located on sub-distal chromosome 2 in the mouse.. Nucleic Acids Research.

[22] Stupp R, Dietrich PY, Ostermann Kraljevic S, Pica A, Maillard I (2002). Promising survival for patients with newly diagnosed glioblastoma multiforme treated with concomitant radiation plus temozolomide followed by adjuvant temozolomide.. J Clin Oncol.

[23] Wang Y, Rudnick PA, Evans EL, Li J, Zhuang Z (2005). Proteome analysis of microdissected tumor tissue using a capillary isoelectric focusing-based multidimensional separation platform coupled with ESI-tandem MS.. Anal Chem.

[24] Maric D, Maric I, Chang YH, Barker JL (2003). Prospective Cell Sorting of Embryonic Rat Neural Stem Cells and Neuronal and Glial Progenitors Reveals Selective Effects of Basic Fibroblast Growth Factor and Epidermal Growth Factor on Self-Renewal and Differentiation.. The Journal of Neuroscience.

